# Dietary host-associated *Bacillus subtilis* supplementation improves intestinal microbiota, health and disease resistance in Chinese perch (*Sinipercachuatsi*)

**DOI:** 10.1016/j.aninu.2023.01.001

**Published:** 2023-01-12

**Authors:** Zhehui Ji, Chuanzhong Zhu, Xinyang Zhu, Sainan Ban, Lijuan Yu, Juan Tian, Lixue Dong, Hua Wen, Xing Lu, Ming Jiang

**Affiliations:** a Yangtze River Fisheries Research Institute, Chinese Academy of Fishery Sciences, Wuhan, China; b Fujian Key Laboratory of Functional Aquafeed and Culture Environment Control, Fujian DBN-HY Aquatic Science and Technology Group Co., Ltd, Zhao’an, China; c Beijing DBN Technology Group Co., Ltd, Beijing, China

**Keywords:** *Bacillus subtilis*, Intestinal microbiota, Intestinal health, Disease resistance, Chinese perch (*Siniperca chuatsi*)

## Abstract

The purpose of this study was to evaluate the potential of a host-associated *Bacillus subtilis* 1-C-7 as a probiotic for Chinese perch (*Siniperca chuatsi*). Four test diets were formulated to contain graded levels of *B. subtilis* 1-C-7 at 0 (CY), 0.85 × 10^8^ (Y1), 0.95 × 10^9^ (Y2) and 0.91 × 10^10^ (Y3) CFU/kg diet. The test fish with initial weight 30.0 ± 1.2 g were fed the 4 test diets with 3 replicates in an indoor water-flow aquaculture system with 12 net cages (40 fish/cage) for 10 wk. At the conclusion of the feeding trial, the probiotic effects of *B. subtilis* on Chinese perch were analyzed based on growth performance, serum biochemical indices, histologic morphology of liver and gut, gut microbiota and the resistance to *Aeromonas hydrophila*. The results showed that the percentage of weight gain had no significant change in the Y1 and Y2 groups (*P* > 0.05) but decreased in the Y3 group compared to that in the CY group (*P* < 0.05). The fish in the Y3 group displayed the highest activity of serum alanine aminotransferase (ALT) and aspartate aminotransferase (AST) among these 4 groups (*P* < 0.05). The fish in the CY group had the highest value of malondialdehyde in the liver (*P* < 0.05) and showed severe nuclear migration and vacuolization of hepatocytes. The morphology indicated that all test fish had poor intestinal health. However, the fish in the Y1 group had a relatively normal intestinal histologic structure. The mid gut microbial diversity analysis showed that dietary *B. subtilis* supplementation increased the abundance of probiotics such as Tenericutes and *Bacteroides*, whereas it reduced the abundance of pernicious bacteria such as *Proteobacteria*, *Actinobacteria*, *Thermophilia* and *Spirochaetes*. The challenge test showed that dietary *B. subtilis* supplementation increased the resistance to *A. hydrophila* in Chinese perch. In conclusion, dietary supplementation of 0.85 × 10^8^ CFU/kg *B. subtilis* 1-C-7 could improve the intestinal microbiota, intestinal health and disease resistance in Chinese perch, but more or excessive supplementation could reduce growth performance and have negative effects on health.

## 1 Introduction

Probiotics are defined as living microbial preparations, with beneficial effects on host by promoting the ecological balance of the intestinal flora (Verschuere et al., 2000). With aquaculture diseases occurring more frequently, greater attention has been paid to the supplementation of exogenous probiotics in diets to improve the immune performance of aquatic animals (Boopathy et al., 2015). Intestinal microbial imbalance is usually the main cause of aquatic animal diseases, so dietary probiotic supplementation has become an effective means to maintain microbial balance (Zorriehzahra et al., 2016). With increasing attention to fish immunization and health, many exogenous probiotics have been widely applied in aquaculture due to their notable antibacterial properties against common pathogenic bacteria and fungi (Lategan et al., 2004; Pieters et al., 2008; Nurhajati et al., 2012; Moosavi-Nasab et al., 2014). However, limitations of probiotics in aquaculture species make it necessary to validate their function before application.

*Bacillus subtilis*, a well-studied exogenous probiotic in aquaculture, can colonize, grow and reproduce in the intestinal tract of aquatic animals, secreting and producing a variety of digestive enzymes and nutritional metabolites (Liu et al., 2015; Shiu et al., 2015; Alan et al., 2016; Xu et al., 2016; Zuenko et al., 2017; Zhang et al., 2021a). Moreover, its supplementation in diets has a strong beneficial effect on many kinds of fish, and no obvious toxicity has been found with an appropriate amount of supplementation (Shiu et al., 2015; Xu et al., 2016; Liu et al., 2018; Adelina et al., 2019; Ahmadifard et al., 2019; Olmos et al., 2020; Wang et al., 2021). The strains of *B. subtilis* in most of these reports were isolated from animals other than the live host. Previous studies have shown that host-associated strains may work better than others (Doan et al., 2020). Therefore, dietary supplementation of *B. subtilis* isolated from the host animal may enable aquaculture producers to get more satisfactory benefits. However, the actual effect of this strain on aquaculture feed still needs to be further studied.

Chinese perch (*Siniperca chuatsi*), a rare freshwater economic carnivorous fish in China, has a high muscle quality and no intramuscular spines, and is rich in highly unsaturated fatty acids (Liang et al., 1998). With increasing promotion of the feed culture mode, the risk of a large-scale epidemic has become an important factor restricting the development of Chinese perch aquaculture as a result of the lack of in-depth research on nutrition and feed (Liu et al., 2017). Thus, in consideration of the beneficial effects of host-associated *B. subtilis*, it is valuable to explore its function on the improvement of fish health and disease resistance, so as to promote the development of Chinese perch culture through feed.

## 2 Materials and methods

### 2.1 Animal ethics

The fish management and sampling were performed following animal care protocols authorized by the Committee on Animal Ethics of Yangtze River Fisheries Research Institute, Chinese Academy of Fisheries Sciences. The protocol number was 2019-41.

### 2.2 *B. subtilis* preparation

The probiotic *B. subtilis* 1-C-7, stored in China Microbial Species Preservation Center (Beijing, China) with the preservation number of CGMCC No. 17348, was isolated from the midgut of Chinese perch. *B. subtilis* 1-C-7 has good antibacterial properties (Appendix Fig. 1) and high production capacities of amylase, lipase and protease (Appendix Fig. 2). The seed liquid, containing 14-h-old *B. subtilis* 1-C-7 with an inoculation amount of 2%, was injected in the fermentation medium with an initial pH of 6.8 prepared as: corn meal (0.5%), soybean cake powder (1%), sucrose (0.4%), fish meal (0.6%), KH_2_PO_4_ (0.1%), FeSO_4_·7H_2_O (0.025%), MgSO_4_·7H_2_O (0.05%), MnSO_4_ (0.024%), CaCO_3_ (0.1%) and defoamers (0.05%).

Fermentation was conducted at 1.11 × *g*, with a ventilation ratio of 4:1 VVM (air volume/culture volume per min) and a pressure of 0.05 MPa. In order to stop fermentation, 15% sterilized brine was added to the broth after 10 h. The viable *B. subtilis* 1-C-7 in the broth was detected in amounts of around 7.5 × 10^10^ CFU/mL. Corn starch (10%, wt/vol) was added into the fermentation liquid of *B. subtilis* 1-C-7 as a protective agent, and the dry powder was collected by spray drying after evenly stirring. The viable count of *B. subtilis* 1-C-7 in the raw powder was 2.0 × 10^10^ CFU/g. The raw powder was diluted 20 times with sodium carboxylmethyl cellulose before the feed preparation.

### 2.3 Experimental diet production

The formulation of the basal diet (CY group) is presented in Table 1. The sodium carboxylmethyl cellulose was replaced by 0.1, 1 and 10 g diluted *B. subtilis* 1-C-7 powder in the basal diet, respectively. The corresponding concentrations of *B. subtilis* 1-C-7 in the 3 experimental diets were 1 × 10^8^ CFU/kg (Y1), 1 × 10^9^ CFU/kg (Y2) and 1 × 10^10^ CFU/kg (Y3) diet, respectively. All dry ingredients were finely ground through a 0.3-mm size sieve, weighed accurately (approximately 0.1 g) and mixed thoroughly. The dry mixture was mixed with fish oil and water in a groove-type mixer (15HW, Shanghai Xingxing Food Machinery Factory, China) for 15 min. The resulting moist mash was passed through a meat chopper machine (TY-432, Shang Hai Tai Yi Machinery, China) fitted with 2 mm in diameter of the moist strands. Then they were crushed into pellets of a desired length (approximately 5 mm). The grinder was equipped with a special water condensing tube to ensure that the temperature did not exceed 50 °C during feed production. Moist pellets were sealed in plastic bags for storage at −20 °C. Prior to feeding, the diets were defrosted at room temperature for about 2 h. The measured concentrations (Zaineldin et al., 2018b) of *B. subtilis* 1-C-7 in the test diets were 0, 0.85 × 10^8^, 0.95 × 10^9^ and 0.91 × 10^10^ CFU/kg diet, respectively.

**Table 1:** Dietary formulation and proximate composition of the basic diet (g/kg).

Item	Content
Ingredients	
Cod fishmeal^1^	600
Fish oil 1	30
Chicken powder 2	80
Peeling soybean meal 3	40
Extruded soybean meal^3^	60
Cassava - starch 4	100
Beer yeast powder 5	40
Sodium carboxymethyl cellulose^6^	20
Vitamin premix^7^	10
Mineral premix^8^	20
Total	1000
Proximate composition (as dry weight)	
Crude protein	520.4
Crude lipid	95.7
Ash	141.2
Gross energy, kJ/g	14.3

1 Fujian Tianma Technology Group Co., Ltd.

2 Shandong Jiahe Biotechnology Co., Ltd.

3 Cofco (Dongguan) Grain and Oil Industry Co., Ltd.

4 Vietnam Investment and Construction Co., Ltd.

5 Yichang Angel Yeast Co., Ltd.

6 Sinopharm Chemical Reagent Co., Ltd.

7 Vitamin premix contained the following amount which were diluted in cellulose (g/kg premix): L-ascorbic acid, 121.2; DL-(tocopheryl acetate, 18.8; thiamin hydrochloride, 2.7; riboflavin, 9.1; pyridoxine hydrochloride, 1.8; niacin, 36.4; Ca-D-pantothenate, 12.7; myo-inositol, 18.8; D-biotin, 0.27; folic acid, 0.68; p-aminobenzoic acid, 18.2; menadione, 1.8; retinyl acetate, 0.73; cholecalciferol, 0.003; cyanocobalamin, 0.003.

8 Mineral premix contained the following ingredients (g/kg premix); MgSO_4_.7H_2_O, 80.0; NaH_2_PO_4_.2H_2_O, 370.0; KCl, 130.0; ferric citrate, 40.0; ZnSO_4_.7H_2_O, 20.0; Ca-lactate, 365.5; CuCl_2_, 0.2; AlCl_3_.6H_2_O, 0.15; KI, 0.15; Na_2_Se_2_O_3_, 0.01; MnSO_4_·H_2_O, 2.0; CoCl_2_.6H_2_O, 1.0.

### 2.4 Fish maintenance

The test fish were obtained from a Chinese perch breeding base (Qingyuan, China). The feeding trial was conducted in the Hai-Kang base of Fujian DBN-HY Aquatic Science and Technology Group (Zhao’an, China; latitude 23.61008° N, longitude 117.24896° E). Indoor water-flow aquarium systems with net cages (1.4 m length × 0.8 m width × 0.8 m water depth per cage) were utilized to culture the fish.

The details of the aquaculture system and fish management procedures were described in our published study (Zhu et al., 2021). Briefly, the fish were maintained in the experimental aquarium system for 7 wk to adapt to the culture environment and artificial feed. The carnivorous fish were fed with live dace (*Cirrhinus molitorella*) in the first 2 wk. Then, these fish were fed with dace that were unable to swim for 3 d, and then fed with dead dace that were killed by 6% sodium chloride solution for 4 d. Over the next 2 wk, the fish were fed with a mixture of dead dace and basal diet at a ratio of 9:1. The portion of basal diet gradually increased by 10% every day until it was completely replaced. In the last 2 wk, the fish were fed with a basal diet to enhance the adaptation to the artificial diet. After acclimation, 480 healthy and uniformly-sized fish (mean body weight = 30.0 ± 1.2 g, *n* = 40) were chosen and divided at random into 4 groups with 12 cages (40 fish per cage). Each group was appointed to 3 net cages and fed with the assigned experimental diet. Fish were hand-fed once a day from 16:00 to 18:00 until apparent satiation was achieved for a period of 10 wk. Each of the wet pellets was manually and slowly fed into the cage to make sure that the fish could eat the feed. Two hours later, uneaten feed was collected using a net and dried to calculate feed intake and feed conversion ratio (FCR). The aquarium system was equipped with a 500-W air pump to provide continuous aeration into each net cage, and freshwater was supplied daily from 08:00 to 18:00 at a speed of about 20 L/min. Approximately 20% of the water was quickly drained at 08:00 to remove fish feces. The water source in the aquaculture system was from groundwater with relatively stable water temperature. During the feeding period, the conditions were as follows: dissolved oxygen >5.0 mg/L, water temperature 23 ± 2.0 °C, salinity <0.1‰, pH of 7.2 ± 0.3, nitrite <0.1 mg/L, ammonia <0.08 mg/L and water transparency was 55 ± 5.0 cm. The experiment was conducted during a natural photoperiod from January to March since a quarter of the roof was transparent.

### 2.5 Sample collection

At the end of the feeding trial, all experimental fish were starved for 24 h before they were batch-weighed and counted to obtain final values of weight gain (WG) and survival (SR). Then, 6 fish from each replicate cage were randomly selected and anesthetized with 75 mg/L tricaine methanesulfonate (Sigma, United States) for subsequent sampling. Three fish in each cage were chosen for the measurement of individual body length and body weight to calculate its condition factor (CF). Another 6 fish from each cage were used for the collection of blood samples by puncturing the caudal vein with a 2-mL syringe. The blood samples were clotted at 4 °C for 4 h, and centrifuged at 1000 × *g* for 10 min at 4 °C, to collect serum for biochemistry analysis. Subsequently, 3 fish from each replicate cage were dissected on ice to determine their liver and viscera, and hepatosomatic index (HSI) and viscerosomatic index (VSI) were calculated accordingly. After that, the liver and midgut of each fish were put into 2 cryopreservation tubes separately, quickly frozen in liquid nitrogen, and stored at −80 °C to analyze oxidative stress and digestive enzyme activity. Parts of the frozen midgut were used for the determination of intestinal flora. Other parts of the liver and midgut were placed in 4% formaldehyde for the histology analysis.

### 2.6 Sample analysis

The detection of serum biochemical indices in fish was performed as previously described (Yu et al., 2018). Briefly, the activities of alanine aminotransferase (ALT), alkaline phosphatase (ALP) and aspartate transaminase (AST), and the concentrations of total cholesterol (TCHO), albumin (ALB), total protein (TP), low density lipoprotein (LDL) and glucose (GLU) in serum were measured by an automatic biochemical analyzer (Sysmex-800, Sysmex Corporation, Kobe, Japan).

Liver samples were weighed accurately (about 0.6 g per sample) and put into a 10 mL homogenate tube. Nine times the volume of 0.86% normal saline was added into the homogenate tube to the ratio of weight (g) to volume (mL) = 1:9. Under the conditions of an ice water bath, the samples were immediately cut up with ophthalmic scissors and homogenized quickly (1,500 × *g*, 10 s/time, interval 30 s, 3 to 5 times, block in ice water). The homogenates were centrifuged (4,500 × *g*, 10 min, 4 °C), the upper impurities were removed with a cotton swab, and then the supernatant was carefully separated with a 200-μL pipettor for analysis. Commercial reagent kits from Nanjing Jiancheng Bioengineering Institute (Nanjing, China) were used to determine the levels of malondialdehyde (MDA) and the activity of amylase (AMS), lysozyme (LZM), acid phosphatase (ACP) and alkaline phosphatase (ALP) in the liver. The content of MDA was determined using the thiobarbituric acid method (Cat. No. A003-1), AMS activity was determined using the starch-iodine color method (Cat. No. C016-1-1). The activities of ACP and ALP were determined using microtitration assay (Cat. No. A060-2-1 and A059-2-2), and LZM activity was determined using the turbidimetry method (Cat. No. A050-1-1).

The liver and gut kept in 4% formaldehyde were made into paraffin sections by Wuhan Servicebio Technology Co., Ltd. (Wuhan, China) and then stained with hematoxylin and eosin (H&E).

The V3-V4 regions of the bacteria 16S ribosomal DNA gene were amplified by PCR using primers 338F 5′-ACTCCTACGGGAGGCAGCA-3′ and 806R 5′-GGACTACHVGGGTWTCTAAT-3′, where barcode was designed. Purified amplicons were pooled in equimolar and paired-end sequences (2 × 250 bp) on an Illumina MiSeq platform according to the standard protocols. Raw fastq files were demultiplexed, quality filtered and analyzed with QIIME and R packages (v3.2.0). The raw fastq data have been uploaded to the database of National Center for Biotechnology Information (NCBI), and the BioProject ID is PRJNA838943. Venn analysis was used to demonstrate the species that were shared and unique among the 4 groups. Nonmetric Multidimensional Scaling analysis (NMDS) was used to analyze the structure of microbial communities in the different groups. Linear discriminant analysis Effect Size (LEfSe) was used to identify bacterial lineages whose frequencies varied significantly depending on treatment variables.

### 2.7 Challenge test

The pathogenic bacteria *Aeromonas hydrophila* were derived from the State Key Laboratory of Direct-fed Microbial Engineering (DBN). After finishing the feeding experiment, 20 experimental fish from each cage were randomly chosen and injected intraperitoneally with 1 mL of bacterial suspension per 100 g fish. The injected concentration of bacteria was approximately 4 × 10^7^ CFU/mL, based on a previous challenge test. Then, the injected fish were returned to the same cage for 7-d observation. The dead fish were recorded each day and the total mortality rate was calculated as follows: mortality rate (%) = 100 × numbers of dead fish/numbers of inoculated fish.

### 2.8 Statistical analysis

All analyses were conducted by using SPSS 25.0 (Chicago, United States). Data were presented as mean ± SD (standard deviation of the mean). Levene's test was applied to the homogeneity of variances. One-way analysis of variance (ANOVA) was performed to test the effect of dietary *B. subtilis.* Tukey's test was used to compare the mean values of treatments when overall differences appeared significant (*P* < 0.05).

## 3 Results

### 3.1 Growth performance

The growth parameters are presented in Table 2. The fish in the Y3 group had the lowest percentage of weight gain (WG), specific growth rate (SGR) and condition factor (CF) among the 4 groups (*P* < 0.05). However, there were no significant differences among the CY, Y1 and Y2 groups. Feed conversion ratio showed the opposite trend to percentage of weight gain. Dietary *B. subtilis* 1-C-7 supplementation had no significant impact on hepatosomatic index (HSI) and viscerosomatic index (VSI). The survival (SR) showed a slight increase from 91.7% to 99.2%, but there was no significant difference among groups.

**Table 2:** Growth performance of mandarin fish fed test diets containing difference levels of *Bacillus subtilis* 1-C-7 for 10 wk.

Group^1^	CY	Y1	Y2	Y3
IBW^2^, g	31.5 ± 0.8	29.5 ± 1.1	30.3 ± 1.7	31.2 ± 1.4
FBW^3^, g	83.4 ± 4.4^b^	83.4 ± 2.2^b^	80.7 ± 5.4^ab^	74.8 ± 3.4^a^
WG^4^, %	165.0 ± 14.7^b^	182.7 ± 18.0^b^	166.0 ± 6.9^b^	140.0 ± 4.8^a^
SGR^5^, %/day	1.39 ± 0.08^ab^	1.48 ± 0.09^b^	1.40 ± 0.04^ab^	1.25 ± 0.03^a^
FCR^6^	1.03 ± 0.05^a^	0.96 ± 0.06^a^	1.08 ± 0.09^a^	1.38 ± 0.14^b^
HSI^7^, %	1.58 ± 0.22	1.64 ± 0.19	1.47 ± 0.45	1.43 ± 0.14
VSI^8^, %	10.1 ± 1.8	9.8 ± 0.6	9.7 ± 0.4	9.0 ± 0.9
CF^9^, g/cm^3^	2.27 ± 0.07^b^	2.19 ± 0.03^b^	2.26 ± 0.03^b^	2.11 ± 0.01^a^
SR^10^, %	91.7 ± 8.0	95.0 ± 5.0	96.7 ± 3.8	99.2 ± 1.4

IBW = initial mean body weight; FBW = final mean body weight; WG = percentage of weight gain; SGR = specific growth rate; FCR = feed conversion ratio; HSI = hepatosomatic index; VSI = viscerosomatic index; CF = condition factor; SR = survival.

^a, b^ Mean values within a row with different superscripts differ significantly at *P* < 0.05. Data were presented mean ± SD, *n* = 3.

1 The diets CY, Y1, Y2, and Y3 contained 0, 0.85 × 10^8^, 0.95 × 10^9^ and 0.91 × 10^10^ CFU/kg *Bacillus subtilis* 1-C-7 diet (as dried weight), respectively.

2 IBW = initial total fish weight per tank/initial fish number per tank.

3 FBW = final total fish weight per tank/final fish number per tank.

4 WG = 100 × (FBW – IBW)/IBW.

5 SGR = 100[(Ln final body weight − Ln initial body weight)/days].

6 FCR = feed intake per tank/(total final fish weight – total initial fish weight + dead fish); FCR were calculated by dried matter.

7 HSI = 100 × (liver weight/body weight).

8 VSI = 100 × (viscera weight/body weight).

9 CF = 100 × [body weight/(body length)^3^].

10 SR = 100 × (final fish number/initial fish number).

### 3.2 Serum biochemical indices

Parts of serum biochemical indices were significantly influenced by dietary *B. subtilis* 1-C-7 supplementation (Table 3). The fish in the Y3 group showed higher activities of ALT and AST and concentrations of TCHO compared those in the CY group. The indices such as ALP, GLU and TP were not influenced by dietary *B. subtilis* 1-C-7 supplementation.

**Table 3:** Serum biochemical indices of mandarin fish fed diets containing difference levels of *Bacillus subtilis* for 10 wk.^1^

Item	CY	Y1	Y2	Y3
ALP, U/L	53.3 ± 5.3	49.5 ± 3.4	46.5 ± 6.2	48.3 ± 5.3
ALT, U/L	10.0 ± 2.6^a^	14.7 ± 3.1^ab^	19.0 ± 2.1^b^	19.33 ± 1.0^b^
ALB, g/L	8.8 ± 1.0^b^	7.3 ± 0.6^a^	8.0 ± 0.0^ab^	8.8 ± 1.0^b^
AST, U/L	108.7 ± 18.4^a^	101.3 ± 4.7^a^	120.0 ± 19.2^b^	159.3 ± 11.0^b^
GLU, mmol/L	8.7 ± 2.7	10.0 ± 2.0	11.0 ± 2.6	8.5 ± 0.8
TCHO, mmol/L	7.3 ± 0.7^a^	7.3 ± 1.1^a^	8.2 ± 1.0^ab^	10.5 ± 3.0^b^
TP, g/L	49.3 ± 2.6	46.0 ± 1.0	46.8 ± 1.7	51.3 ± 3.3

ALP = alkaline phosphatase; ALT = alanine aminotransferase; ALB = albumin; AST = aspartate aminotransferase; GLU = glucose; TCHO = Total cholesterol; TP = total protein.

^a, b^ Mean values within a row with different superscripts differ significantly at *P* < 0.05. Data were presented mean ± SD, *n* = 3.

1 The diets CY, Y1, Y2, and Y3 contained 0, 0.85 × 10^8^, 0.95 × 10^9^ and 0.91 × 10^10^ CFU/kg *B. subtilis* 1-C-7 diet (as dried weight), respectively.

### 3.3 Oxidative stress and digestive enzyme activity analysis

Oxidative stress and digestive enzyme activity are presented in Table 4. The MDA content in the liver was significantly decreased by dietary *B. subtilis* 1-C-7 supplementation. The fish fed the diet without *B. subtilis* 1-C-7 had the highest value of MDA, while the fish in the Y2 group had the lowest value of MDA among the 4 groups. Furthermore, the value of MDA in the Y3 group was markedly higher than that in the Y2 group. The ALP activity in the liver was elevated with the increasing of dietary *B. subtilis* 1-C-7 supplementation. The activity of lysozyme in the liver was not affected by dietary *B. subtilis* 1-C-7 supplementation.

**Table 4:** Liver biochemical indices of mandarin fish fed diets containing difference levels of *Bacillus subtilis* 1-C-7 for 10 wk.^1^

Item	CY	Y1	Y2	Y3
MDA, μmol/g protein	60.8 ± 17.9^c^	35.5 ± 4.0^b^	13.2 ± 6.8^a^	35.4 ± 9.0^b^
AMS, U/g protein	174.7 ± 10.5^a^	190.4 ± 16.5^ab^	197.4 ± 16.6^ab^	206.3 ± 18.8^b^
ACP, U/g protein	296.0 ± 51.4	258.7 ± 67.1	239.8 ± 35.1	206.0 ± 51.0
LZM, μg/g protein	14.6 ± 3.02	16.6 ± 0.8	15.8 ± 1.6	16.0 ± 0.5
ALP, U/g protein	61.0 ± 9.0^a^	68.3 ± 7.2^a^	101.0 ± 1.4^b^	141.0 ± 27.0^c^

MDA = malondialdehyde; AMS = amylase; ACP = acid phosphatase; LZM = lysozyme; ALP = alkaline phosphatase.

^a,b,c^ Mean values within a row with different superscripts differ significantly at *P* < 0.05. Data were presented mean ± SD, *n* = 3.

1 The diets CY, Y1, Y2, and Y3 contained 0, 0.85 × 10^8^, 0.95 × 10^9^ and 0.91 × 10^10^ CFU/kg *B. subtilis* 1-C-7 diet (as dried weight), respectively.

### 3.4 Liver and intestinal morphology

**Fig. 1:**
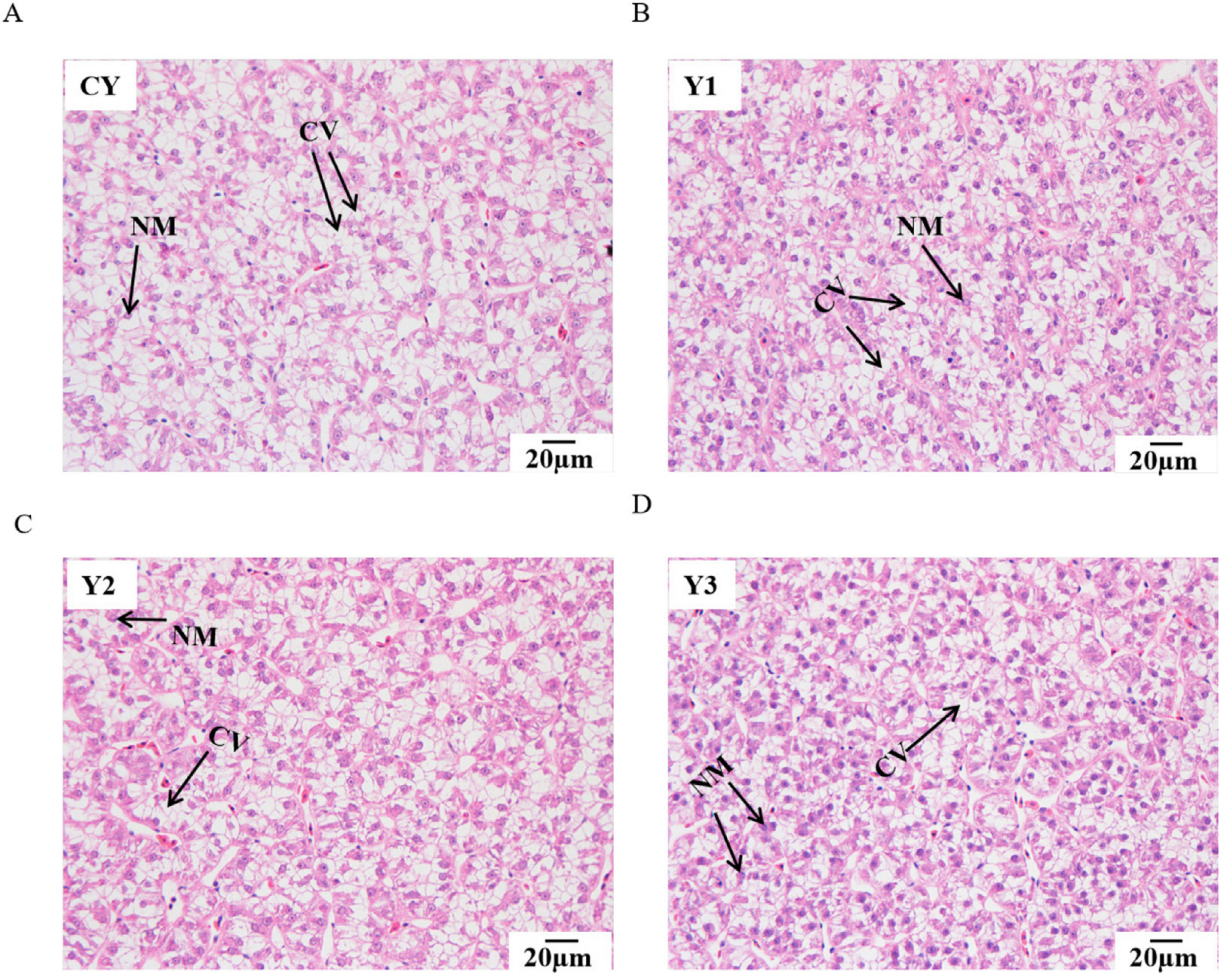
Histologically-observed liver of mandarin fish fed with test diets (magnification 400× ). NM = nuclear migration; CV = cellular vacuolization. The diets CY (A), Y1(B), Y2 (C), and Y3 (D) contained 0, 0.85 × 10^8^, 0.95 × 10^9^ and 0.91 × 10^10^ CFU/kg *B. subtilis* 1-C-7 diet (as dried weight), respectively.

**Fig. 2:**
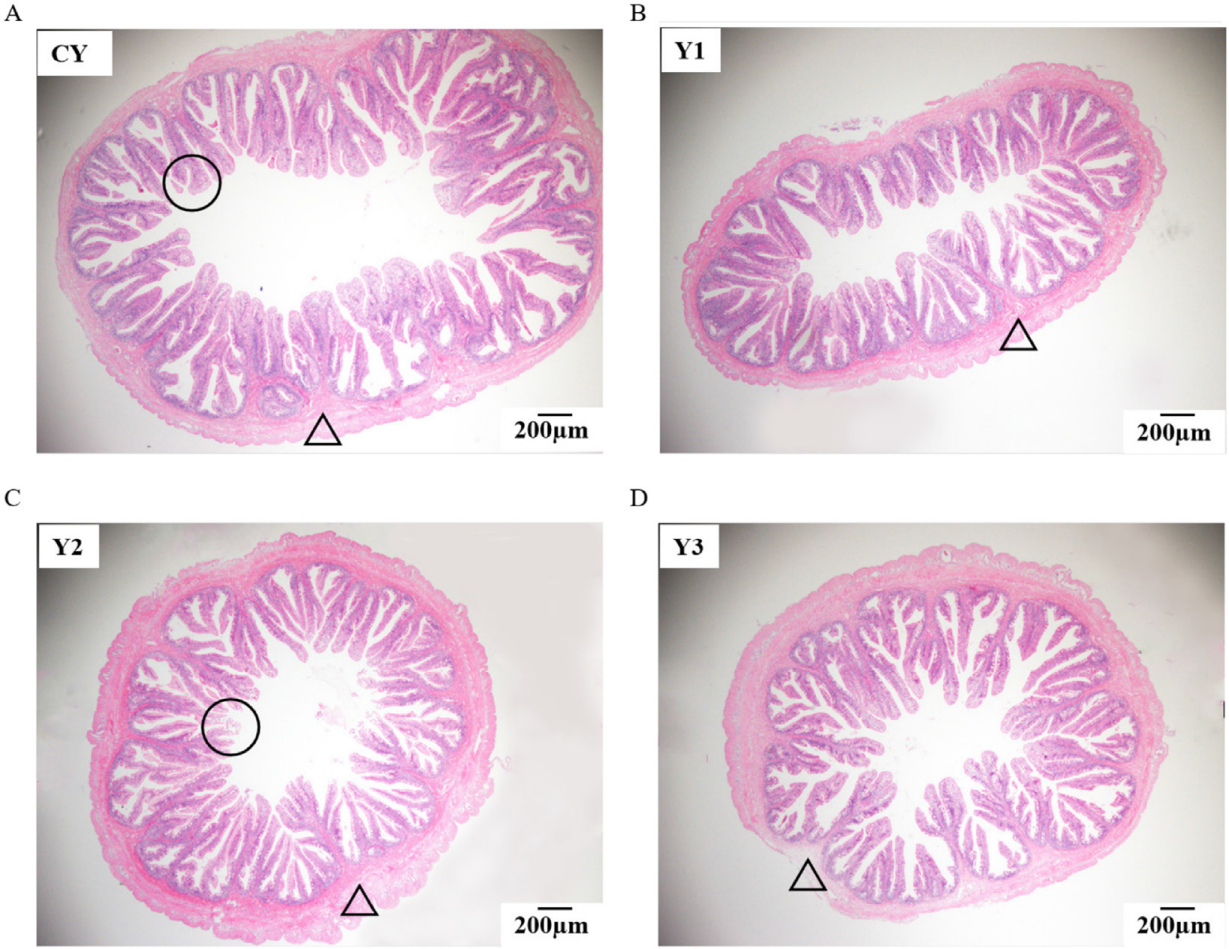
Histologically-observed mid-gut of mandarin fish fed with test diets (magnification 40× ). Triangles refer to intestinal wall hyperplasia; Circles refer to intestinal mucous membrane shedding. The diets CY (A), Y1(B), Y2 (C), and Y3 (D) contained 0, 0.85 × 10^8^, 0.95 × 10^9^ and 0.91 × 10^10^ CFU/kg *B. subtilis* 1-C-7 diet (as dried weight), respectively.

The H&E staining sections of the liver and gut are shown in Fig. 1, Fig. 2. The fish in the control group showed severe nuclear migration and vacuolization of hepatocytes. This liver health status was obviously improved in the Y1 and Y2 groups, but deteriorated in the Y3 group (Fig. 1 and Appendix Fig. 3). The morphology indicated that all the fish in the 4 groups had poor intestinal health. However, the fish in the Y1 group had a relatively normal intestinal histologic structure (Fig. 2 and Appendix Fig. 4).

### 3.5 **Microbial diversity analysis**

**Fig. 3:**
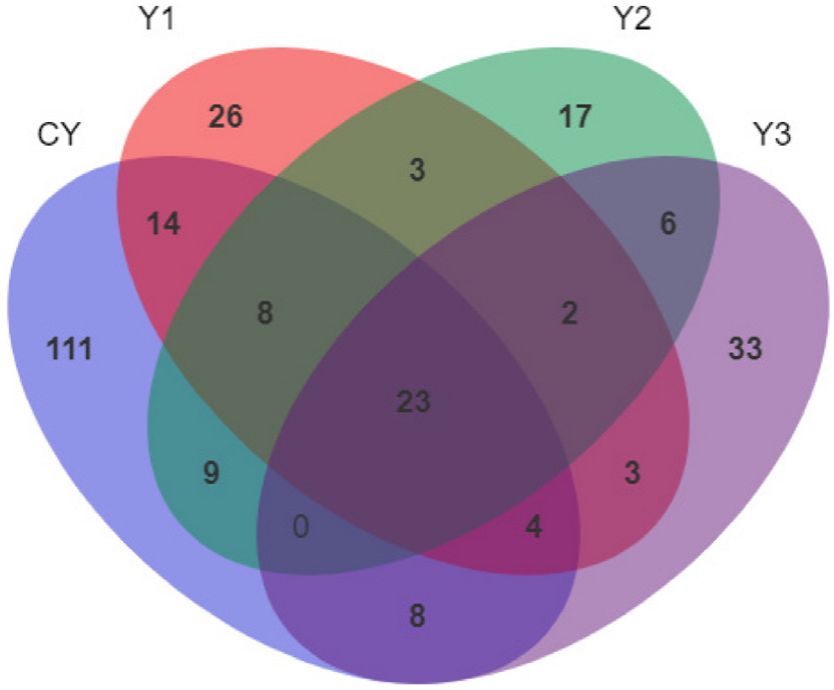
Venn diagram demonstrating the distribution of OTU shared by mandarin fish fed with test diets. The diets CY, Y1, Y2, and Y3 contained 0, 0.85 × 10^8^, 0.95 × 10^9^ and 0.91 × 10^10^ CFU/kg *B. subtilis* 1-C-7 diet (as dried weight), respectively.

The Venn diagram demonstrating the distribution of operational taxonomic units (OUT) shared is shown in Fig. 3. After regression with 97% similarity of the high-quality sequences of the test groups, 177, 83, 68, and 79 OTU were obtained, respectively. There were 111, 26, 17 and 33 unique OTU in the CY, Y1, Y2 and Y3 groups, respectively. The Alpha diversity analysis is shown in Table 5. In comparison to the CY group, the indices of Chao1, Species, Simpson, and Shannon were significantly decreased in the Y1, Y2 and Y3 groups.

**Table 5:** The number of sequences analyzed, estimated OTU richness (Species and Chao), and diversity index (Shannon and Simpson) for 16S rRNA libraries of mid-gut in mandarin fish fed with test diets.^1^

Item	CY	Y1	Y2	Y3
Chao	85.52 ± 9.08^b^	39.9 ± 6.76^a^	34.32 ± 4.17^a^	38.4 ± 6.26^a^
Species	82.23 ± 8.27^b^	38.7 ± 6.53^a^	33.67 ± 4.15^a^	37.5 ± 5.96^a^
Shannon	0.72 ± 0.09^b^	0.33 ± 0.03^a^	0.37 ± 0.05^a^	0.37 ± 0.05^a^
Simpson	0.16 ± 0.02^b^	0.07 ± 0.00^a^	0.08 ± 0.01^a^	0.08 ± 0.01^a^

^a,b^ Mean values within a row with different superscripts differ significantly at *P* < 0.05. Data were presented mean ± SD, *n* = 3.

1 The diets CY, Y1, Y2, and Y3 contained 0, 0.85 × 10^8^, 0.95 × 10^9^ and 0.91 × 10^10^ CFU/kg *B. subtilis* 1-C-7 diet (as dried weight), respectively.

**Fig. 4:**
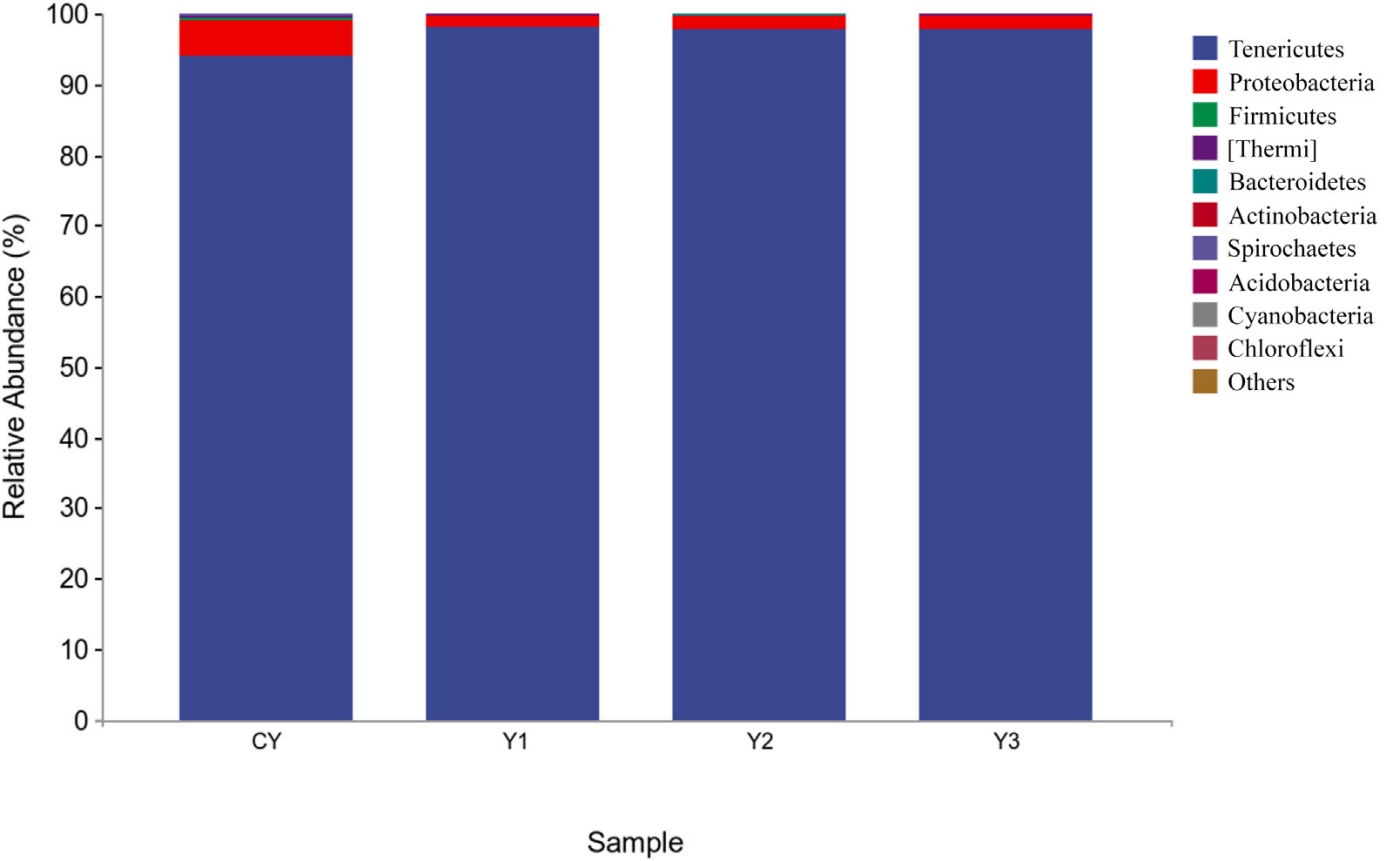
Relative abundance of intestinal bacteria at the phylum level in mandarin fish fed with test diets. The diets CY, Y1, Y2, and Y3 contained 0, 0.85 × 10^8^, 0.95 × 10^9^ and 0.91 × 10^10^ CFU/kg *B. subtilis* 1-C-7 diet (as dried weight), respectively.

**Fig. 5:**
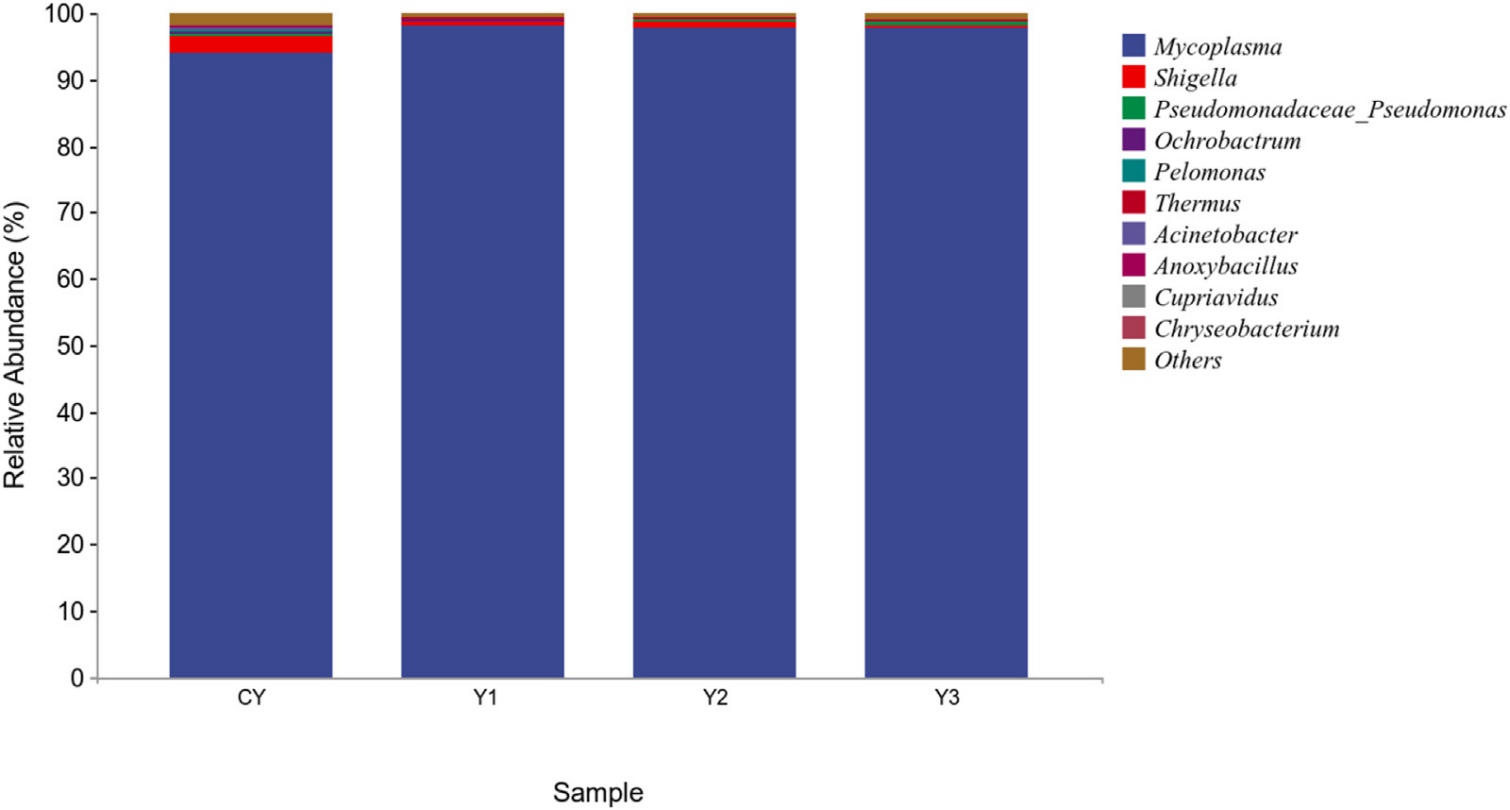
Relative abundance of intestinal bacteria at the genus level in mandarin fish fed with test diets. The diets CY, Y1, Y2, and Y3 contained 0, 0.85 × 10^8^, 0.95 × 10^9^ and 0.91 × 10^10^ CFU/kg *B. subtilis* 1-C-7 diet (as dried weight), respectively.

The relative abundance of intestinal bacteria at the phylum level is shown in Fig. 4. Tenericutes and Proteobacteria were the main dominant bacteria in all groups. The Y1, Y2 and Y3 groups showed higher abundances of Tenericutes and lower abundances of Proteobacteria and Firmicutes compared to the CY group (Appendix Table 1). The abundance of *Thermophilia*, *Actinobacteria*, *Spirochaetes*, *Acidobacteria* and *Cyanobacteria* in the Y1, Y2 and Y3 groups was obviously lower than that in the CY group and the highest abundance of Bacteroidetes was found in the Y2 group. The relative abundance of intestinal bacteria at the genus level is shown in Fig. 5. *Mycoplasma* was the main dominant bacteria in all groups. The highest relative abundance of *Mycoplasma* was in the Y1 group and the lowest was in the CY group (Appendix Table 2). The abundance of *Shigella, Acinetobacter* and *Cupriavidus* was significantly decreased compared with the CY group.

**Fig. 6:**
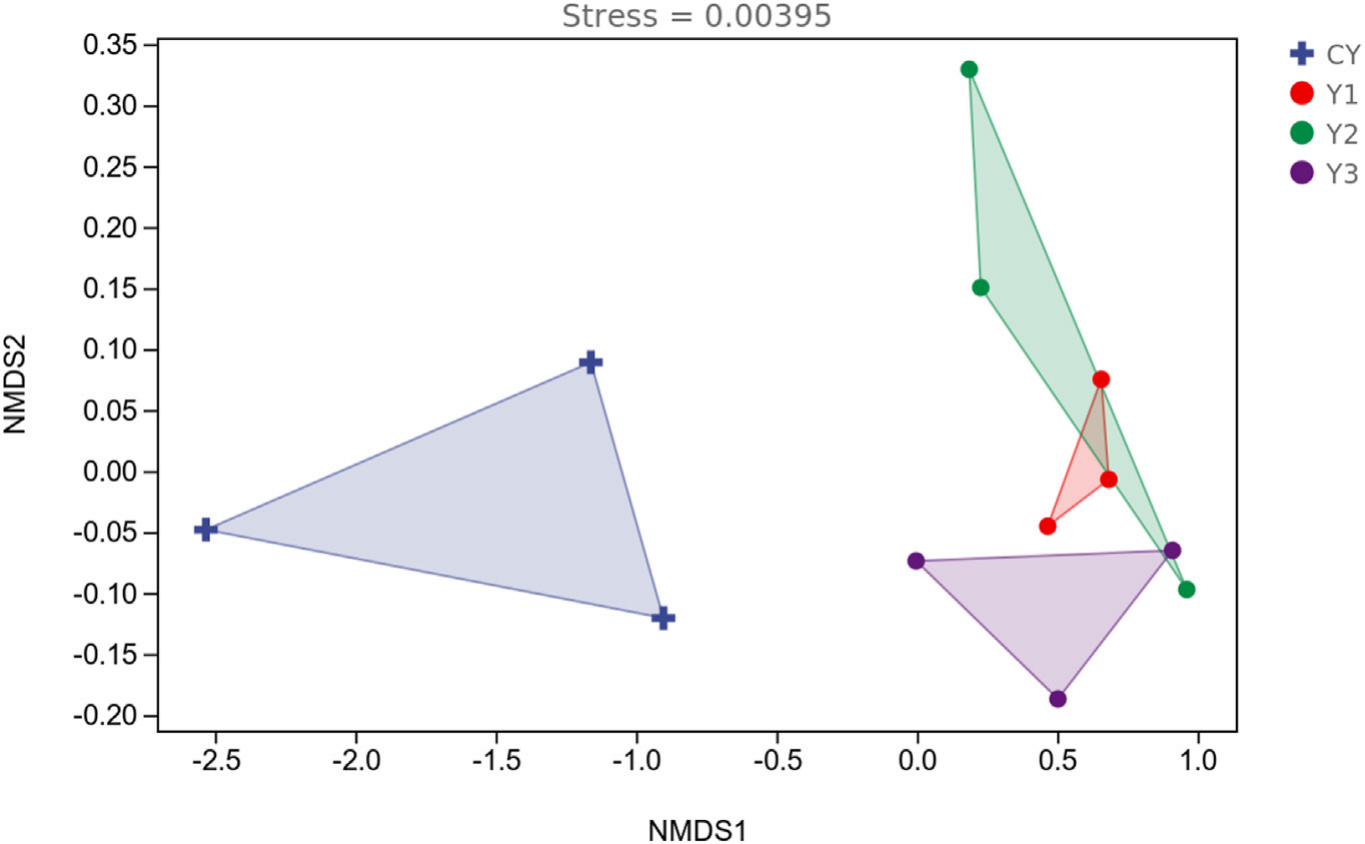
Nonmetric Multidimensional Scaling analysis (NMDS) of bacterial community structures in mandarin fish fed with test diets. The diets CY, Y1, Y2, and Y3 contained 0, 0.85 × 10^8^, 0.95 × 10^9^ and 0.91 × 10^10^ CFU/kg *B. subtilis* 1-C-7 diet (as dried weight), respectively.

**Fig. 7:**
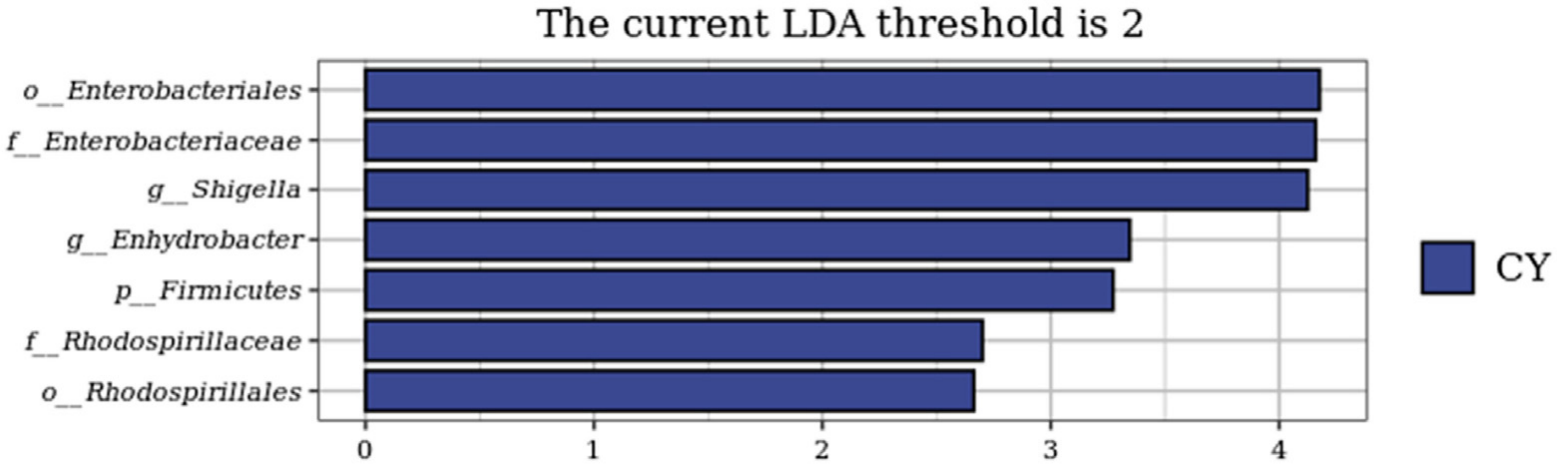
Linear discriminant analysis Effect Size (LEfSe) bar of group CY. The diets CY contained 0 CFU/kg *B. subtilis* 1-C-7 diet (as dried weight).

The NMDS of bacterial community structures is shown in Fig. 6. The CY group was separated from the other 3 groups of samples, while the samples from groups Y1, Y2, and Y3 were cross-clustered with each other. The LEfSe bar is shown in Fig. 7. The LDA scores of *Enterobacteriaceae*, *Enterobacteriales* and *Shigella* in the CY group were greater than other groups, which indicated that these microbes played an important role in differentiating the groups. Thus, these microbes could be used as the landmark species for the differences between the groups.

### 3.6 Challenge test

**Fig. 8:**
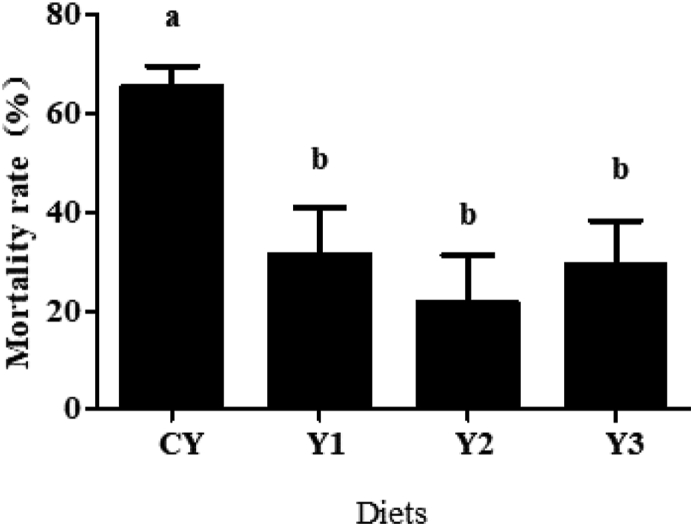
Mortality rate following a 7-day *Aeromonas hydrophila* challenge of mandarin fish fed with difference dietary *B. subtilis* 1-C-7. Values within the same period with different superscript letters are significantly different (*P* < 0.05). The diets CY, Y1, Y2 and Y3 contained 0, 0.85 × 10^8^, 0.95 × 10^9^ and 0.91 × 10^10^ CFU/kg *B. subtilis* 1-C-7 diet (as dried weight), respectively.

The mortality rate of fish in the challenge test is presented in Fig. 8. The mortality rate in the Y1, Y2 and Y3 groups decreased significantly compared with that in the CY group.

## 4 Discussion

*B. subtilis* can produce several different exogenous enzymes that provide large amounts of micronutrients, which contribute to the decomposition of nutrients (Liu et al., 2012; Zuenko et al., 2017). The effect of dietary *B. subtilis* supplementation on growth performance has been found to be different in aquatic animals (Liu et al., 2012; Mohapatra et al., 2012; Sedaghat et al., 2012; Zaineldin et al., 2018a; Xue et al., 2020). In the present study, dietary supplementation of *B. subtilis* 1-C-7 had no significant effect on the growth of Chinese perch at the concentration of 0.85 × 10^8^ CFU/kg and 0.95 × 10^9^ CFU/kg. This result could be due to the unchanged FCR and amylase activity. However, *B. subtilis* 1-C-7 overdose inhibited the growth of Chinese perch, as the excessive addition of *B. subtilis* caused a significant increase in amylase activity, which was presumed to be caused by the amylase secreted by the exogenous *B. subtilis*. Chinese perch have poor tolerance to dietary carbohydrate (Zhang et al., 2021b). Increased amylase activity may improve the digestibility of starch in mandarin fish, leading to an increase in the level of digestible carbohydrates, resulting in liver injury and decreased growth performance. Similar results were obtained in other studies (Zhu et al., 2021; Akter et al., 2019).

Adequate dietary supplementation of *B. subtilis* can improve disease resistance and antioxidant capacity of aquatic animals (Taoka et al., 2006; Aly et al., 2008), and this conclusion is also supported by the decrease of the mortality rate in the challenge test. A similar unpublished study conducted by our lab indicated that dietary host *B. subtilis* could enhance the secretion of antibacterial substances, such as aromatic amino acids and their derivatives, and reduce the proliferation of challenge bacteria in the intestine of tilapias and thus increase the survival rate of tilapia after infection with *Streptococcus agalactiae*. The increase of survival of mandarin fish after challenge with *A. hydrophila* may also be attributed to the secretion of antimicrobial substances by *B. subtilis*.

MDA is known to indicate the lipid peroxidation rate or reflect tissue peroxidation damage; the decrease in MDA content may suggest that dietary *B. subtilis* supplementation could improve the antioxidant capacity of Chinese perch. Serum biochemical parameters could reflect fish nutritional status, and are often used to determine its metabolism and physiological state. The TCHO level in serum is a crucial indicator that reflects lipid metabolism. In the present experiment, serum TCHO content was significantly lower in the Y3 group than that in the CY group, suggesting that *B. subtilis* 1-C-7 might affect lipid metabolism in mandarin fish. Serum ALT and AST are important amino acid transferases that are common in fish hepatocyte mitochondria. These 2 parameters are also known to reflect the status of liver health in fish. In this study, fish fed the Y2 and Y3 diets had higher AST and ALT activities than fish in the other groups. These results further evidence that a relatively high dose of *B. subtilis* 1-C-7 may suppress fish growth performance and health.

The liver is an important organ for lipid metabolism. When ingested, *B. subtilis* secretes large amounts of amylase and lipase, thus reducing the pressure on liver lipid metabolism which may decrease inflammation of the liver. These results suggested that dietary supplementation of 0.85 × 10^8^ CFU/kg *B. subtilis* 1-C-7 could improve disease resistance and intestinal health in Chinese perch, but dietary supplementation of 0.95 × 10^9^ CFU/kg and 0.91 × 10^10^ CFU/kg *B. subtilis* 1-C-7 might be harmful to health.

In fish, the gut microbiota play an indispensable role in the life cycle, and its abundance and structure were closely related to the vital activity (Kosiewicz et al., 2014). In this study, dietary *B. subtilis* 1-C-7 supplementation (Y1, Y2 and Y3) increased the abundance of Tenericutes, and reduced the abundance of pernicious bacteria such as *Proteobacteria*, *Actinobacteria*, *Thermophilia* and *Spirochaetes*. Bacteroidetes in the gut can produce polysaccharide hydrolases that can effectively degrade dietary fiber to produce monosaccharides and short-chain fatty acids, and the increase of their abundance might provide a boost to the body metabolism (Bradlow, 2014). Proteobacteria and Actinobacteria, common pathogenic bacteria, are closely related to intestinal inflammation, immune disorders and other diseases, and the increase of their abundance might help to balance the intestinal flora structure to a certain extent (Mohapatra et al., 2012). In addition, the increase of abundance of *Mycoplasma* and decrease of abundance of *Shigella* and *Anoxybacillus* in the present study might indicate an improvement in health status and prevention of some intestinal diseases (Bozzi et al., 2021). However, excessive supplementation of *B. subtilis* 1-C-7 (Y3) increased the abundance of common pathogenic bacteria such as *Pseudomonas*, indicating a diminution of beneficial effects or a deleterious effect of dietary *B. subtilis* 1-C-7, thus potentially damaging the intestinal environment and inhibiting growth. These results suggested that in Chinese perch, dietary supplementation of 0.85 × 10^8^ CFU/kg *B. subtilis* 1-C-7 could improve the intestinal bacterial community structure, but dietary supplementation of 0.91 × 10^10^ CFU/kg *B. subtilis* 1-C-7 might be harmful to intestinal microbiota.

## 5 Conclusion

In conclusion, dietary supplementation of *B. subtilis* 1-C-7 improved the intestinal microbiota, intestinal health and *A. hydrophila* disease resistance of Chinese perch. However, excessive supplementation of *B. subtilis* 1-C-7 reduced growth performance and had negative effects on health. Based on the data of this experiment, the optimal dosage of *B. subtilis* 1-C-7 in the diet is approximately 1 × 10^8^ CFU/kg for Chinese perch.

## Author contributions

**Zhehui Ji:** Data curation, Writing – original draft preparation, Visualization. **Chuanzhong Zhu:** Conceptualization. **Xinyang Zhu:** Funding acquisition. **Sainan Ban:** Methodology. **Xing Lu:** Data curation. **Lijuan Yu**, **Juan Tian** and **Lixue Dong**: Investigation. **Hua Wen**: Resources. **Ming Jiang:** Project administration, Writing- Reviewing and Editing.

## Declaration of competing interest

We declare that we have no financial and personal relationships with other people or organizations that can inappropriately influence our work, and there is no professional or other personal interest of any nature or kind in any product, service and/or company that could be construed as influencing the content of this paper.

## Data Availability

All data are available from the corresponding author by request.
