# Supplementary material for: Dietary host-associated Bacillus subtilis supplementation improves intestinal microbiota, health and disease resistance in Chinese perch (Sinipercachuatsi)
Source: Anim Nutr. 2023 Jan 12;13:197–205. doi: 10.1016/j.aninu.2023.01.001 (PMC10300398; doi:10.1016/j.aninu.2023.01.001)
Supplement: Multimedia component 1 [file mmc1.docx]

**Appendix Tables & Figures**

| **Appendix Table 1** Relative abundance of intestinal bacteria at the phylum level in Mandarin fish fed with test diet. | | | | |
| --- | --- | --- | --- | --- |
| Group | CY | Y1 | Y2 | Y3 |
| Tenericutes, % | 94.00±1.67^a^ | 98.25±0.29^b^ | 97.85±1.01^b^ | 97.94±1.00^b^ |
| Proteobacteria, % | 4.93±1.14^b^ | 1.50±0.23^a^ | 1.72±0.80^a^ | 1.74±0.98^a^ |
| Firmicutes, % | 0.40±0.13^b^ | 0.06±0.03^a^ | 0.09±0.06^a^ | 0.14±0.05^a^ |
| Thermi, % | 0.27±0.16 | 0.12±0.06 | 0.11±0.08 | 0.12±0.05 |
| Bacteroidetes, % | 0.07±0.05 | 0.03±0.02 | 0.16±0.24 | 0.02±0.01 |
| Actinobacteria, % | 0.13±0.03^b^ | 0.04±0.02^a^ | 0.07±0.06^b^ | 0.02±0.02^a^ |
| Spirochaetes, % | 0.12±0.20 | 0.00±0.00 | 0.00±0.00 | 0.00±0.00 |
| Acidobacteria, % | 0.05±0.08 | 0.00±0.00 | 0.00±0.00 | 0.00±0.01 |
| Cyanobacteria, % | 0.01±0.01 | 0.00±0.00 | 0.00±0.00 | 0.01±0.01 |
| Chloroflexi, % | 0.01±0.01 | 0.00±0.00 | 0.00±0.00 | 0.00±0.01 |
| Others, % | 0.01±0.02 | 0.00±0.01 | 0.00±0.00 | 0.00±0.01 |

^a, b^ Mean values within a row with different superscripts differ significantly at *P* < 0.05. Data were presented mean ± SD, *n* = 3.

The diets CY, Y1, Y2 and Y3 contained 0, 0.85 × 10^8^, 0.95 × 10^9^ and 0.91 × 10^10^ CFU/kg *Bacillus subtilis* 1-C-7 diet (as dried weight), respectively.

| **Appendix Table 2** Relative abundance of intestinal bacteria at the genus level in Mandarin fish fed with test diet. | | | | | |
| --- | --- | --- | --- | --- | --- |
| Group | CY | Y1 | Y2 | Y3 |  |
| *Mycoplasma* % | 94.01±1.67^a^ | 98.25±0.29^b^ | 97.85±1.01^b^ | 97.94±1.00^b^ |  |
| *Shigella* % | 2.65±0.51^b^ | 0.52±0.41^a^ | 1.03±0.47^a^ | 0.14±0.15^a^ |  |
| *Pseudomonas* % | 0.12±0.02 | 0.07±0.02 | 0.05±0.04 | 0.54±0.86 |  |
| *Ochrobactrum* % | 0.32±0.18 | 0.14±0.06 | 0.12±0.06 | 0.13±0.10 |  |
| *Pelomonas* % | 0.28±0.11 | 0.13±0.08 | 0.12±0.07 | 0.13±0.06 |  |
| *Thermus* % | 0.27±0.15 | 0.12±0.05 | 0.12±0.08 | 0.12±0.04 |  |
| *Acinetobacter* % | 0.24±0.09^b^ | 0.15±0.07^ab^ | 0.02±0.01^a^ | 0.07±0.04^a^ |  |
| *Anoxybacillus* % | 0.11±0.07 | 0.02±0.02 | 0.02±0.02 | 0.02±0.01 |  |
| *Cupriavidus* % | 0.09±0.03^b^ | 0.03±0.02^ab^ | 0.02±0.02^a^ | 0.03±0.01^ab^ |  |
| *Chryseobacterium* % | 0.00±0.00 | 0.01±0.01 | 0.13±0.23 | 0.01±0.01 |  |
| Others % | 1.93±0.66^b^ | 0.57±0.30^a^ | 0.52±0.12^a^ | 0.87±0.49^ab^ |  |

^a, b^ Mean values within a row with different superscripts differ significantly at *P* < 0.05. Data were presented mean ± SD, *n* = 3.

The diets CY, Y1, Y2 and Y3 contained 0, 0.85 × 10^8^, 0.95 × 10^9^ and 0.91 × 10^10^ CFU/kg *Bacillus subtilis* 1-C-7 diet (as dried weight), respectively.

**
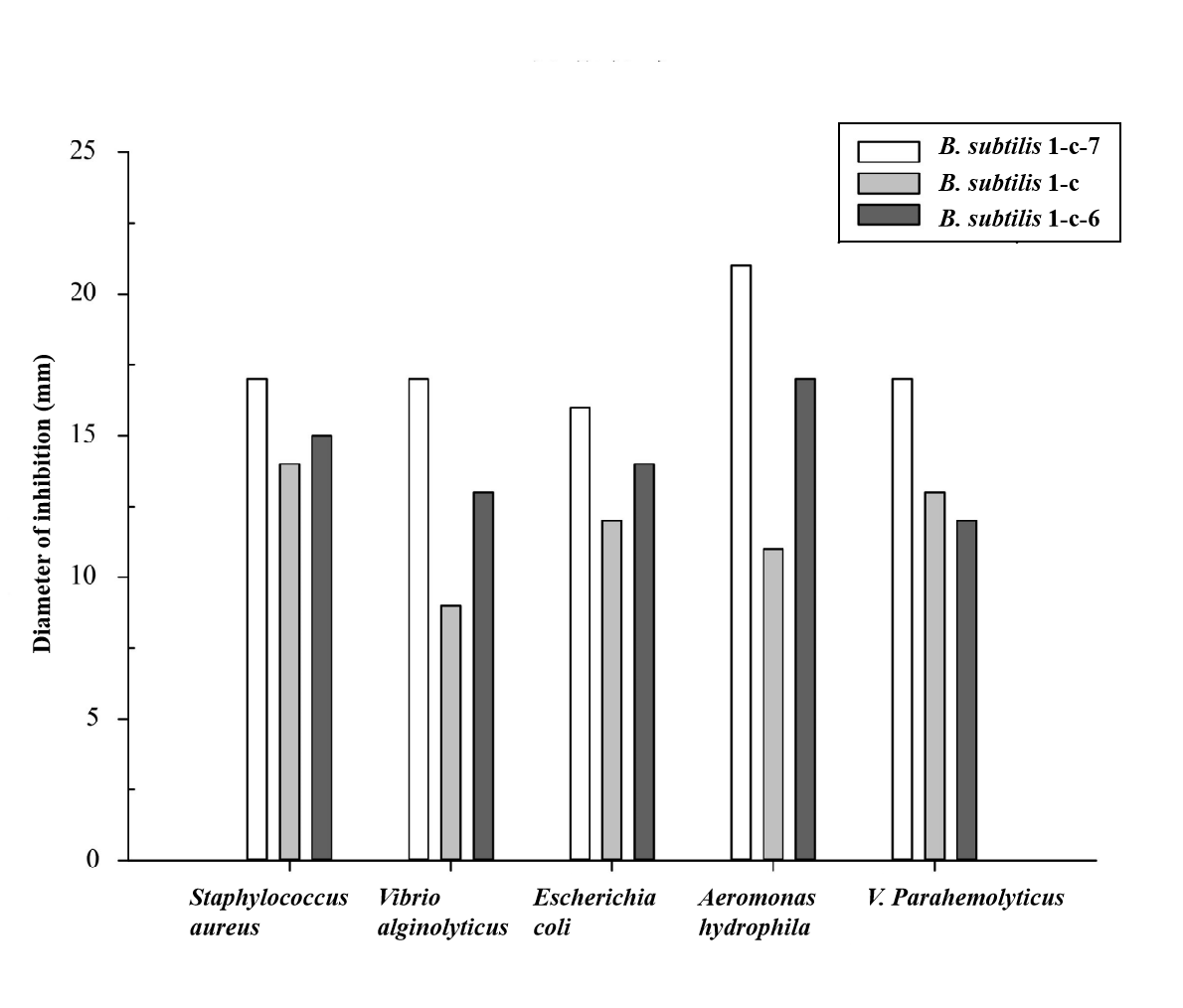
**

**Appendix Fig. 1** Bacteriostatic effects of the *Bacillus subtilis* 1-c7 compared with *Bacillus subtilis* 1-c-6 and *Bacillus subtilis* 1-c. Diameter of inhibition by *Bacillus subtilis* 1-c-7 against fungal pathogens: *Staphylococcus aureus*; *Vibrio alginolyticus*; *Escherichia coli*; *Aeromonas hydrophila*; *V. Parahemolyticus.*


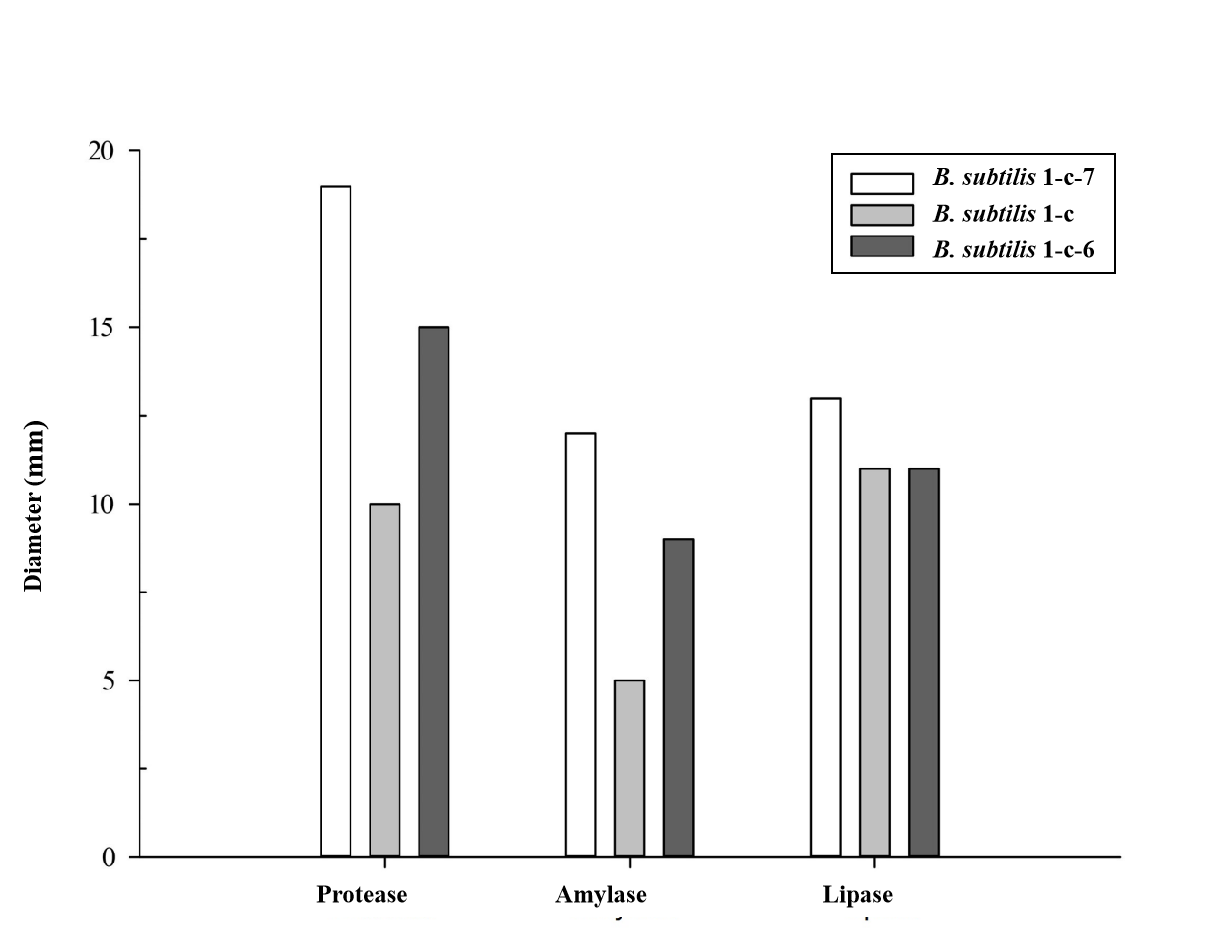


**Appendix Fig. 2** Enzyme production capacity of the *Bacillus subtilis* 1-c-7 compared with *Bacillus subtilis* 1-c-6 and *Bacillus subtilis* 1-c.


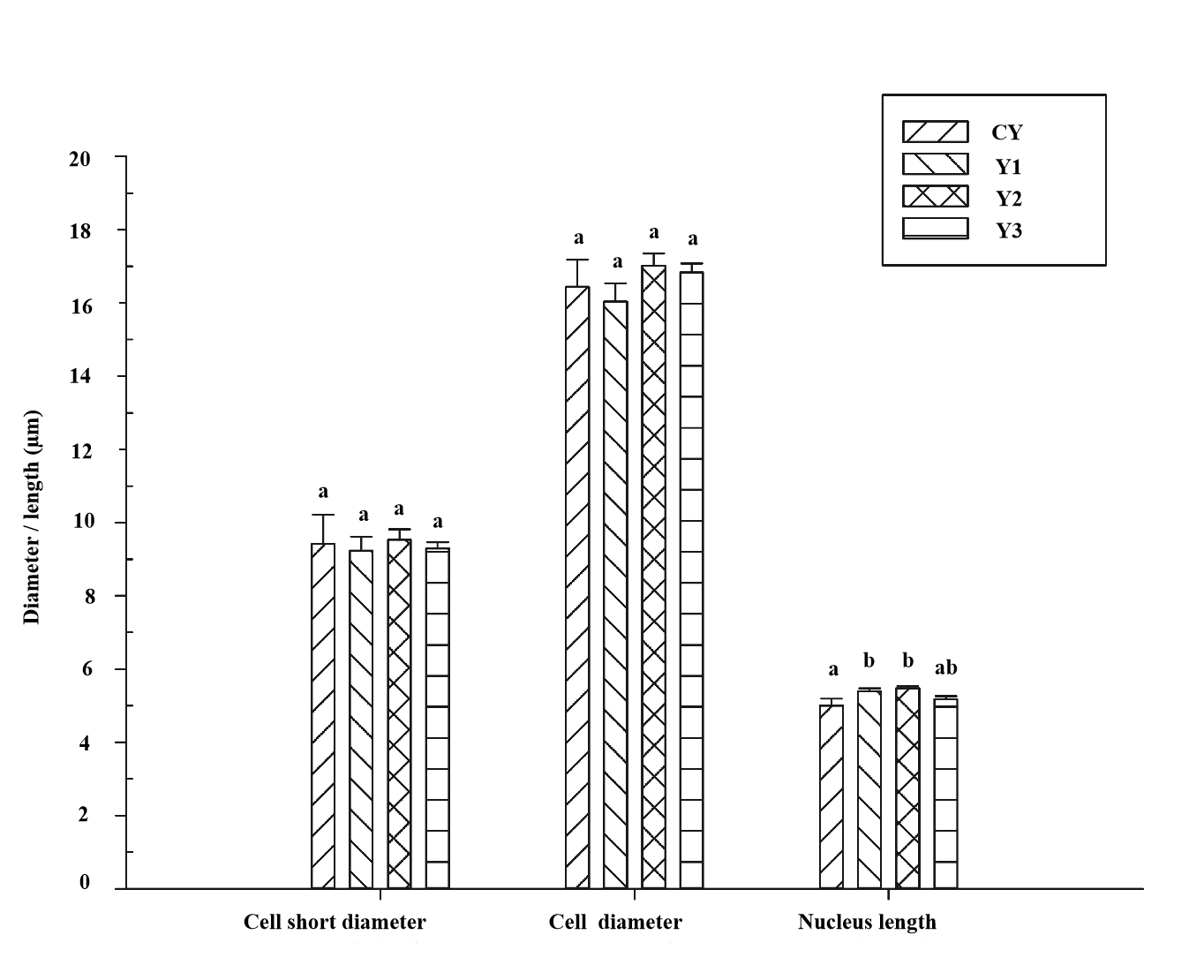


**Appendix Fig. 3** Liver cell morphological indicators of Mandarin fish fed with difference dietary *Bacillus subtilis* 1-C-7. Values within the same period with different superscript letters are significantly different (*P* < 0.05). The diets CY, Y1, Y2 and Y3 contained 0, 0.85 × 10^8^, 0.95 × 10^9^ and 0.91 × 10^10^ CFU/kg *Bacillus subtilis* 1-C-7 diet (as dried weight), respectively.


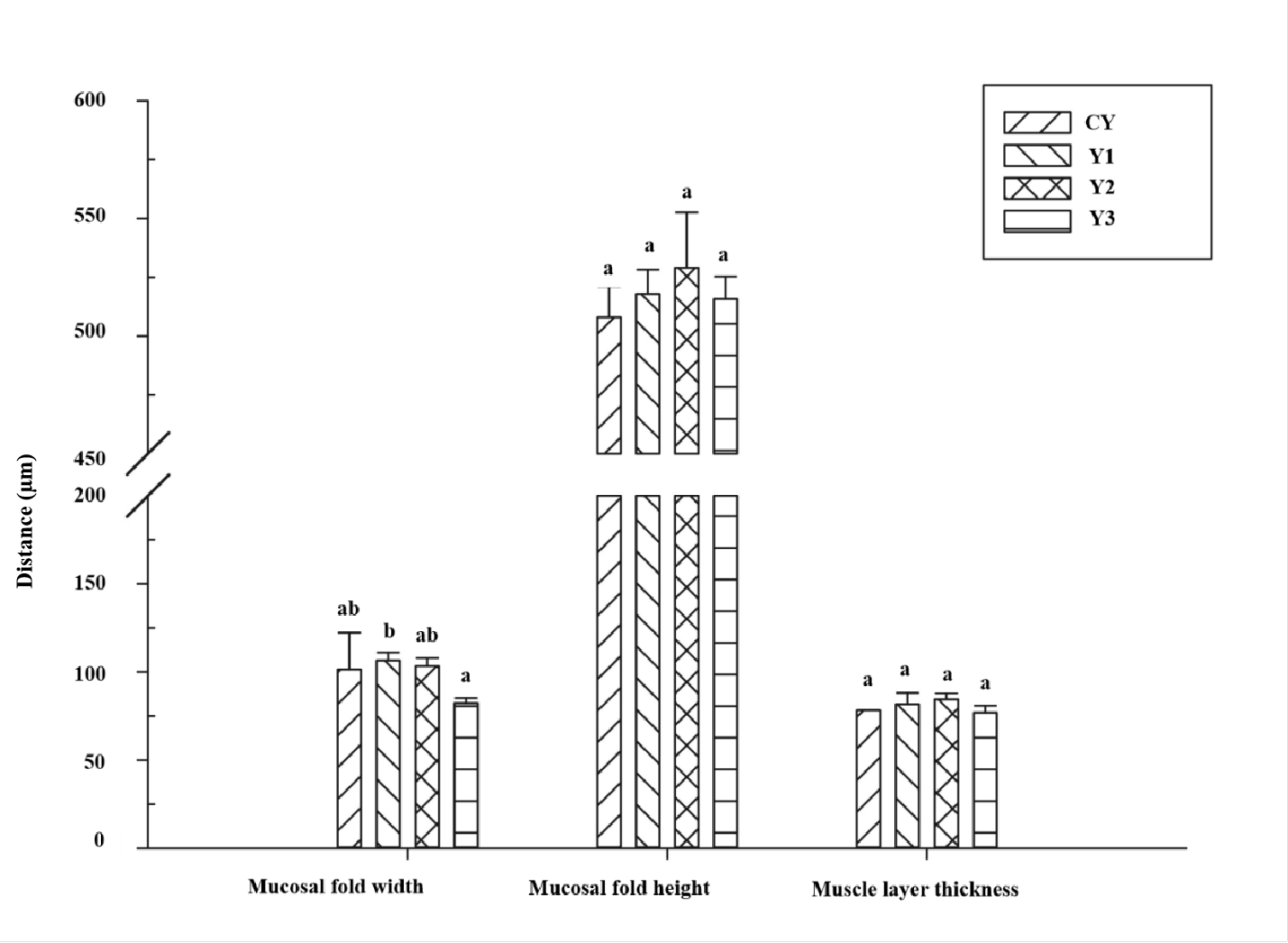


**Appendix Fig. 4** Intestinal morphology indicators of Mandarin fish fed with difference dietary *Bacillus subtilis* 1-C-7. Values within the same period with different superscript letters are significantly different (*P* < 0.05). The diets CY, Y1, Y2 and Y3 contained 0, 0.85 × 10^8^, 0.95 × 10^9^ and 0.91 × 10^10^ CFU/kg *Bacillus subtilis* 1-C-7 diet (as dried weight), respectively.
